# 92418 Molecular imaging of the tumor microenvironment to predict response to combination treatment with immunotherapy in triple negative breast cancer

**DOI:** 10.1017/cts.2021.455

**Published:** 2021-03-30

**Authors:** Tiara S. Napier, Chanelle L. Hunter, Patrick N. Song, Benjamin M. Larimer, Anna G. Sorace

**Affiliations:** University of Alabama at Birmingham

## Abstract

ABSTRACT IMPACT: Insights from this project will provide clinical guidance in treatment of immunotherapy in triple negative breast cancer and identify early imaging biomarkers of treatment response. OBJECTIVES/GOALS: Significant research that addresses monitoring and predicting patient response of triple negative breast cancer (TNBC) to immunotherapy is needed. Using positron emission tomography (PET) imaging to probe the tumor microenvironment (hypoxia, T-cell activation), we aim to predict early response to immunotherapy for in mouse models of TNBC tumors. METHODS/STUDY POPULATION: Female Balb/c mice with 4T1-luciferase mammary carcinoma cell tumors were administered paclitaxel (PTX; 10 mg/kg), anti-PD1 (200 µg), both, or vehicle (saline) intraperitoneally. Treatment was given on days 0, 2, and 5 for cohort 1 (n=16) who underwent granzyme B specific (GZP) PET imaging (T-cell activation) and days 0, 2, 5, and 8 for cohort 2 (n=12) who underwent [18F]-fluoromisonidazole (FMISO)-PET imaging (hypoxia). Bioluminescence (BLI) imaging and caliper measurements were performed to track tumor size changes at multiple timepoints and tumors were collected for histological validation on day 20. Mean standard uptake value (SUVmean) was calculated as percent of day 0, and statistical analyses were performed with unpaired t-tests and Wilcoxon-rank sum tests. RESULTS/ANTICIPATED RESULTS: Non-responders to treatment had a significantly higher tumor volume compared to responders starting on day 6 (p<0.05). Although no significant differences in BLI between control and single-agent therapies were found, BLI data revealed that treatment with combination PTX and anti-PD1 significantly decreased viability signal between days 3 and 6 (p=0.04). SUVmean from GZP-PET was over 250% higher in responders compared to non-responders by day 6 (p=0.03). SUVmean from FMISO-PET was 80% less in responders compared to nonresponders, indicating less tumor hypoxia (p=0.04). DISCUSSION/SIGNIFICANCE OF FINDINGS: Non-invasive PET imaging of the tumor microenvironment can provide data on T cell activation and hypoxic response predicting response to combination immunotherapy and chemotherapy. Utilizing advanced imaging to understand biologically distinct features of the TNBC tumor microenvironment can aid in personalizing anti-cancer therapies.

